# Construction Notes

**Published:** 1895-12-14

**Authors:** 


### Construction Notes.

**New Hospital for Burton on-Trent.**

The opening of the new hospital for infectious diseases cases took place early this month. The institution consists of a series of blocks, situated on a sheltered terrace on the northern extremity of the Outwood hills. There are six buildings, but two only are used for the reception and treatment of fever cases. The remainder consists of an administrative block. The total cost, inclusive of the freehold site, is estimated at about £11,000. The buildings were designed and their construction superintended by the borough surveyor, Mr. J. E. Swindlehurst, C.E., and the contract was carried out by Mr. Hodges, of Burton.

